# Regulation of inflammation and immunity in sepsis by E3 ligases

**DOI:** 10.3389/fendo.2023.1124334

**Published:** 2023-07-03

**Authors:** Shasha Shao, Daixing Zhou, Jun Feng, Yanyan Liu, Baturuhu, Huimei Yin, Daqian Zhan

**Affiliations:** ^1^ Department of Emergency Medicine, Tongji Hospital, Tongji Medical College, Huazhong University of Science and Technology, Wuhan, China; ^2^ Department of Critical Care Medicine, Tongji Hospital, Tongji Medical College, Huazhong University of Science and Technology, Wuhan, China; ^3^ Obstetrics and Gynecology Department, Tongji Hospital, Tongji Medical College, Huazhong University of Science and Technology, Wuhan, China; ^4^ Department of Neurosurgery Intensive Care Unit (ICU), People’s Hospital of Bortala Mongol Autonomous Prefecture, Bole, China; ^5^ Department of Emergency Medicine, People’s Hospital of Bortala Mongol Autonomous Prefecture, Bole, China

**Keywords:** E3 ligases, ubiquitination, inflammation, immunity, sepsis

## Abstract

Sepsis is a life-threatening organ dysfunction caused by an abnormal infection-induced immune response. Despite significant advances in supportive care, sepsis remains a considerable therapeutic challenge and is the leading cause of death in the intensive care unit (ICU). Sepsis is characterized by initial hyper-inflammation and late immunosuppression. Therefore, immune-modulatory therapies have great potential for novel sepsis therapies. Ubiquitination is an essential post-translational protein modification, which has been known to be intimately involved in innate and adaptive immune responses. Several E3 ubiquitin ligases have been implicated in innate immune signaling and T-cell activation and differentiation. In this article, we review the current literature and discuss the role of E3 ligases in the regulation of immune response and their effects on the course of sepsis to provide insights into the prevention and therapy for sepsis.

## 1 Introduction

Sepsis is defined as life-threatening organ dysfunction caused by a dysregulated host response to infection and is an important global health problem (1). It is reported that sepsis affects nearly 50 million people annually and accounts for about 11 million deaths per year worldwide (2). The highest incidence has been observed in young children and older adults, with the major causes being respiratory tract and abdominal infections (3). As with an in-depth understanding of sepsis, it has been thought to be mainly induced by the dysregulated immune response. In the early stage of sepsis, systemic activation of the innate immune system results in a severe and persistent inflammatory response characterized by an excessive release of inflammatory cytokines such as IL-1, TNF, and IL-17, collectively known as the “cytokine storm”. If surviving the initial stage of hyper-inflammation, patients frequently transit into a later stage of prolonged immune suppression, which is characterized by markedly impaired innate and adaptive immune function (4). Therefore, attempting to understand the underlying changes that occur in both innate and adaptive immune systems may highlight potential new targets for sepsis.

Ubiquitination is an essential post-translational protein modification and plays a pivotal role in the regulation of various biological processes, including immune responses (5–7). The process of ubiquitination is achieved through a series of enzymatic steps involving a ubiquitin-activating enzyme (E1), a ubiquitin-conjugating enzyme (E2), and a ubiquitin ligase (E3), resulting in the transfer of covalently bound ubiquitin from the E2 protein to a lysine residue on the target protein (8). Ubiquitin is composed of 76 amino acids and contains seven lysine residues (K6, K11, K27, K29, K33, K48, K63), which can be linked to substrate proteins in the form of monomers, branched chains, and linear chains (9–11). In the human genome, there are two E1s, approximately 38 E2s, and over 600 E3 ligases (12–14). Unlike E1 and E2, E3 exhibits structural diversity, which is a prerequisite for the precise selection of substrate proteins (15). Thus, E3 determines the substrate selectivity, specificity and is a key regulator of ubiquitination. Classically, these E3 ligases are classified into three families, RING (Really interesting new gene), HECT (homologous to E6AP C-terminus), and RBR (RING-between RING). In addition, some E3 ligases for ubiquitin-like proteins (Ubls) are considered atypical E3 ligases, such as Ubiquitin-fold modifier1 (UFM1) (16). Because the detailed structure and features of E3 ligases are well illustrated in the other excellent reviews (15, 17), in this review, we will briefly introduce the E3 ligases family and primarily discuss recent progress regarding the roles of E3 ligases in regulating the immune response in sepsis, focusing on innate immune signaling such as pattern recognition receptor (PRR) signaling and T-cell activation and differentiation in sepsis.

## 2 E3 ligase family

### 2.1 RING

The RING E3 ligases can be classified into four different sub-families, including the tripartite motif (TRIM), membrane-associated RING-CH (MARCH), PA-TM-RING, and RING-Ub interaction motif (UIM) families. There are approximately 60 members in the TRIM family, which are involved in a variety of physiological processes such as autophagy, cancer, and immune response (18). The MARCH family consists of 11 members (termed MARCH-1 to 11) that share a similar structure. MARCH family proteins have an N-terminal cytoplasmic tail containing a C4HC3 RING finger (RING-CH finger) motif and two or more transmembrane (TM) domains, except for MARCH7 and MARCH10, which have no predicted TM domains (19). Recent studies have demonstrated that MARCH proteins are critical regulators of immune responses, which act by catalyzing polyubiquitination of various immune receptors or certain organelle membrane-associated components involved in innate immune responses (20, 21). PA-TM-RING is structurally characterized by the presence of a protease-associated (PA) domain, a RING-H2 domain, and a TM domain and has been reported to negatively regulate cell proliferation (22). Some PA-TM-RING E3 ligases like RNF128/GRAIL play a role in T-cell survival, activation, and differentiation (23). The UIM family has so far identified four members, namely RNF114, RNF125, RNF138, and RNF166, which structurally share a C2HC-type zinc finger, UIM-type domain, and the C2H2-type zinc fingers, in addition to the RING domain (24). These family members are likely to be involved in the regulation of T-cell activation (24) and the IFN signaling pathway (25–27).

### 2.2 HECT

There are 28 HECT E3 ligases in humans and can be divided into three major subfamilies based on substrate binding domains: the neuronal precursor cell-expressed developmentally downregulated 4 (NEDD4), HECT, and RLD domain-containing (HERC) and ‘other’ HECTs (28). There are nine members in the NEDD4 family such as NEDD4, ITCH, WWP1, WWP2, SMURF1, and SMURF2 and all of them contain an N-terminal single Ca^2+^-binding C2 domain followed by two, three or four WW domains. The HERC family comprises six members which are characterized by the presence of one or more regulators of chromosome condensation 1 (RCC1) protein-like domains (RLDs) at their N-termini. In addition, some HECT E3 ubiquitin ligases have variable protein–protein interaction domains at their N-termini and do not contain any RLD or WW domains, so they have been broadly grouped into the ‘other’ HECT E3 ligase family (29). The HECT E3 ubiquitin ligases are involved in a myriad of cellular processes and signaling pathways (29). For example, NEDD4 promotes the ubiquitylation and degradation of Casitas B lymphoma-b (Cbl-b), a RING E3 ubiquitin ligase that facilitates the destruction of components of the T-cell receptor (TCR) signaling to initiate the immune response (30). The overexpression of HERC4 facilitates the proliferation of hepatocellular carcinoma (HCC), whereas depletion of HERC4 slows down proliferation and increases the apoptotic rate of HCC (31). SMURF2 and WWP1 induce the downregulation of TGF-β signaling through the degradation of TGF-β receptor via the ubiquitin-proteasome pathway (32–34).

### 2.3 RBR

Fourteen E3 ligases with RBR domains have been identified in humans. The 14 family members of RBR E3 ligases include ARIH1, ARIH2, Ankib1, PARC, Parkinson protein2, RBCK1, RNF14, RNF19A, RNF19B, RNF31, RNF144A, RNF144B, RNF216, and RNF217 (35, 36). RBR E3 ligases are characterized by the RING1-IBR-RING2 motif (13, 37, 38). RING1 has a cross-supported zinc coordination structure, while IBR and RING2 each have two zinc ions that coordinate linearly. Unlike RING E3 ligases, RBR E3 ligases do not directly transfer ubiquitin from E2 ligases to substrate proteins, but form a covalent intermediate with ubiquitin, similar to HECT E3 ligase, then transfers this ubiquitin to the substrate (15, 39–43). Each RBR E3 ligase has its function. For example, ARIH1 is associated with the activation of cellular immune responses and is involved in the regulation of cell proliferation and cycle. ARIH2 negatively regulates NLRP3 inflammasome activation in macrophages (44). RNF216 inhibits macrophage autophagy and is an important factor in autophagy degradation and ubiquitination (45).

### 2.4 Atypical E3 ligases

Almost all E3 ligases are categorized into three types. However, some E3 ligases for Ubls are considered to be atypical E3 ligases. Ubls encompass a family of proteins that share structural and evolutionary relationships with ubiquitin. For example, UFM1 is one of the ubiquitin-like proteins. UFM1 is conjugated to its target proteins by a three-step enzymatic reaction. The UFM1-specific ligase 1 (UFL1) acts as the E3 to recognize its substrate, transfer, and ligate the UFM1 from E2 to the substrate. This process is called UFMylation, and the system is conserved in multicellular organisms. A UFM1 cascade is closely related to human diseases. UfL1 has no sequence homology to any other known E3 ligases for ubiquitin and ubiquitin-like modifiers (15). Furthermore, UfL1 is the only one E3 identified to date for UFM1. Small ubiquitin-like modifier (SUMO) is another member of ubiquitin-like proteins that are covalently attached to target proteins as a post-translational modification (termed SUMOylation). Similar to ubiquitin, conjugation is achieved through a cascade of activities that are catalyzed by E1 activating enzymes, E2 conjugating enzymes, and E3 ligases. In this system, RanBP2 is an SUMO E3 ligase and is required for the efficient and proper transfer of SUMO from E2 to a target protein. Numerous proteins have been identified as SUMO target proteins including the important regulatory proteins c-Jun, p53, histone, and histone deacetylase (46–48). While the ubiquitin system includes more than 600 E3 ligases, only a few E3 ligases have been reported so far for SUMO.

## 3 The E3 ligase and sepsis

### 3.1 Roles of E3 ligase in sepsis by regulating the innate immune response

Innate immunity is the host’s first line of defense and is intended to prevent infection. When pathogens invade, PRRs expressed on innate immune cells, such as macrophages and neutrophils, recognize pathogen-associated molecular patterns (PAMPs) and initiate the host immune response. The PRR family of receptors includes Toll-like receptors (TLRs), and cytosolic receptors, e.g, nucleotide-binding oligomerization domain (NOD)-like receptors (NLRs) and retinoic acid-inducible gene I (RIG-I)-like receptors (RLRs). PRRs activation results in the activation of the NF-κB (nuclear factor kappa-B) and MAPK (Mitogen-activated protein kinase) signaling pathways, leading to the production of pro-inflammatory cytokines, chemokines, and interferons (IFNs). These inflammatory mediators initiate an immune response and begin the recruitment of immune cells to the site of infection. During sepsis, these signaling pathways are abruptly upregulated and result in the release of cytokines, chemokines, and damage-associated molecular patterns (DAMPs), which serve as potent mediators for excessive systemic inflammation and immune suppression. Ubiquitination is crucial for the induction of an adequate but confined immune response. Multiple studies have shown several E3 ligases are crucial regulators in innate immune signaling (49–51).

#### 3.1.1 Role of E3 ligase in TLR signaling

**Figure 1:**
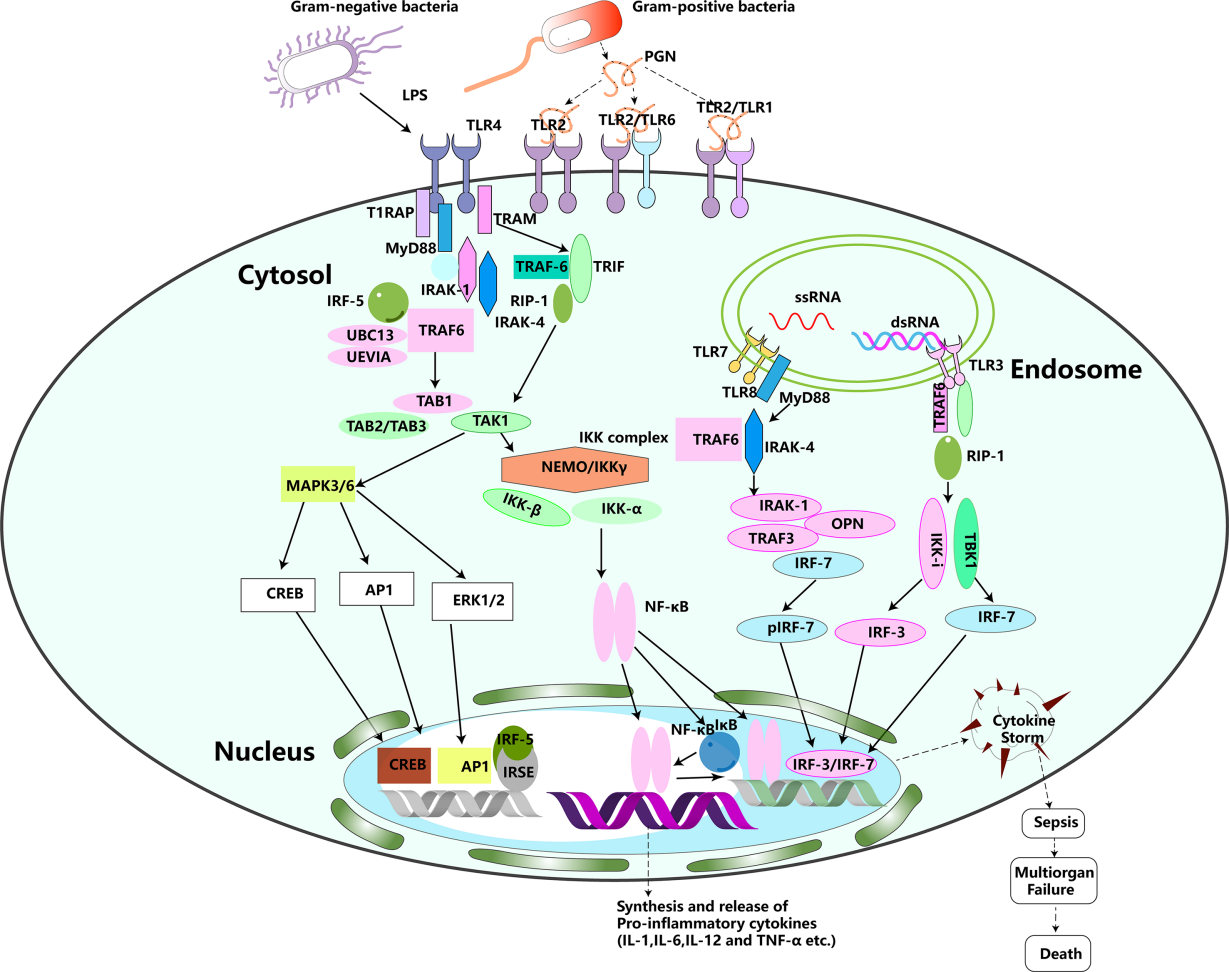
TLR signaling pathways.

TLRs are well-defined PRRs responsible for pathogen recognition and induction of innate immune responses. They can recognize both the external PAMPs and the internal DAMPs (52, 53). To date, at least 11 human TLRs have been identified, which are expressed on various types of immune and non-immune cells, such as macrophages, monocytes, dendritic cells, lymphocytes, endothelial and epithelial cells (54). Depending on their cellular localization and respective PAMP ligands, TLRs are largely divided into two subgroups. One group is composed of TLR1, TLR2, TLR4, TLR5, TLR6, and TLR10, which are expressed on cell surfaces and recognize microbial membrane components such as lipids, lipoproteins, and proteins; the other group is composed of TLR3, TLR7, TLR8, and TLR9, which are expressed exclusively in intracellular vesicles including lysosomes, endosomes, phagosomes and endolysosomes, where they recognize microbial nucleic acids (55, 56). TLRs are type I transmembrane proteins with ectodomains containing leucine-rich repeats that mediate the recognition of PAMPs; transmembrane domains; and intracellular Toll–interleukin 1 (IL-1) receptor (TIR) domains required for downstream signal transduction (57). Upon activation by PAMPs or DAMPs, TLRs form dimers and trigger the downstream signaling through the specific adaptors, i.e., MyD88 (myeloid differentiation factor 88), TIRAP (TIR domain-containing adaptor protein), TRIF (TIR domain-containing adaptor inducing IFN- β mediated transcription factor), SARM (sterile-α and heat-armadillo-motif-containing protein), and TRAM (TRIF related adaptor molecule) (54). TLR signaling can be further divided into two distinct but convergent pathways: MyD88-dependent and TRIF-dependent pathways. (**Figure 1**). The MyD88-dependent pathway is activated by all TLRs except TLR3. MyD88 recruits IL-1 receptor-associated kinase (IRAKs) and TNF receptor-associated factor 6 (TRAF6), which finally leads to the nuclear translocation of pro-inflammatory transcription factor NF-κB. The TRIF-dependent pathway is utilized by TLR3 and TLR4. It induces type I interferon and inflammatory cytokines through the transcription factor interferon regulatory factor 3 (58). Although TLRs are essential for protective immunity against infection, however, excessive TLR activation disrupts the immune homeostasis by sustained pro-inflammatory cytokines and chemokine production and consequently contributes to the development and progression of many diseases, such as sepsis, Alzheimer’s disease, and diabetes-type 1 (59–61). Both experimental models of sepsis and septic human patients display significantly up-regulated TLR expression in various organs. Activation of TLR signaling was reported to cause cytokine storm during sepsis. TLR2 and 4 are the two sponsors to the pathogenesis of sepsis (55, 60, 62–65).Thus, manipulation of TLR signaling pathways has been predicted to be an effective therapeutic strategy for sepsis.

Some E3 ligases have been shown to negatively regulate TLR-induced signaling. For example, Guillamot found that the E3 ligase Speckle-type BTB–POZ protein (SPOP) controls the resolution of systemic inflammation by triggering MyD88 degradation (66). In the absence of SPOP, systemic inflammation proceeded in an unresolved manner, and the sustained response in the hematopoietic stem cells, which proliferate and divert their differentiation toward the myeloid lineage, resulted in a lethal phenotype reminiscent of hyper-inflammatory syndrome or sepsis (66, 67). TRIM13, one of the TRIM family members, is also shown to be associated with the overexpression of TRAF6 and the production of cytokines and inflammatory mediators by enhancing the activity of NF-κB (68). Another E3 ligase Cbl-b has a crucial role in acute inflammation by negatively regulating TLR4 signaling. In a model of polymicrobial sepsis, loss of Cbl-b expression accentuates acute lung inflammation and significantly increases the sepsis-induced release of inflammatory cytokines and chemokines and reduces survival. In the study, Cbl-b was found to control TLR4 signaling by downregulating TLR4 expression or preventing the association of TLR4 and MyD88 (69).

It is well known that the TRIF-dependent pathway plays a key role in the pathogenesis of sepsis by mediating the production of TNF-α, IL-1, IFN-γ, and chemokines. Studies have shown that E3 ligase TRIM family members are related to TRIF post-translational modification. For example, TRIM38 negatively regulates TLR3-mediated IFN-β signaling by targeting TRIF degradation (70). TRIM32^-/-^or TRIM8^-/-^ mice produced higher levels of serum inflammatory cytokines and were more sensitive to infection-induced septic shock and death (71, 72). Further studies confirmed that TRIM32 negatively regulates TLR3/4-mediated immune response by targeting TRIF to TAX1BP1-mediated selective autophagy degradation (71). TRIM8 catalyzes K6- and K33-linked polyubiquitination of TRIF, and the TRIF-anchored polyubiquitination chains impede the assembly of TRIF-associated complexes and thus terminate TLR3/4-mediated inflammation (72). WWP2, a member of HECT E3 ligases, is also involved in TLR signaling by inducing the ubiquitination of TRIF. Loss of function study showed that WWP2 −/− mice exhibited increased pro-inflammatory factors and susceptibility to death to poly (I: C)-induced activation of TLR3 (73). HECT E3 ligase HECTD3 can promote IFN-I production and sepsis by catalyzing ubiquitination of TRAF3(TNF receptor-associated factor family), which is reported to mediate TRIF-dependent type I IFN production (74). In addition, HECTD3 also promotes the dissemination of bacteria (74). NEDD4l is a member of the NEDD4 family, belonging to HECT E3 ligases. It promotes LPS-induced IFN-I by catalyzing the K29-linked ubiquitination of TRAF3. Deficiency of NEDD4l impairs the anti-infection immune response *in vitro* and *in vivo* (75). In addition to the E3 ligases described above, many other E3 ligases can regulate the TLR signaling pathways to alter inflammation or immune response in sepsis (**Table 1**).

**Table 1:** E3 ligases act on substrates or signaling pathways to regulate TLR signaling.

E3	Family	Substrate/signal pathway	Function	References
SPOP	RING E3	MyD88	Inhibition of TLR4-mediated NF-κB activation and inflammatory cytokine production	(66, 67)
TRIM28	RING E3	IRF7	Inhibition of IRF7-inducedIFNsand ISRE promoter activity.	(76)
TRIM13	RING E3	TRAF6	Promoting K9-linked polyubiquitin chains for TRAF6 ubiquitination to promote NF-κB activity and thus potentiated activation of TLR2-mediated immune responses	(68)
Cbl-b	RING E3	TLR4	Preventing hyperactivation of NF-κB	(69)
TRIM38	RING E3	TRIF	negatively regulates TLR3-mediated type I interferon signaling by targeting TRIF	(70)
TRIM32	RING E3	TRIF	negatively regulates TLR3/4-mediated immune responses by targeting TRIF to TAX1BP1-mediated selective autophagic degradation.	(71)
TRIM8	RING E3	TRIF	Promoting the polyubiquitination of TRIF at the K6-linked and K33 junctions, impeding the assembly of TRIF-associated complexes and thereby terminating TLR3/4-mediated inflammation.	(72)
TRAF1	RING E3	TRIF-dependent pathway	Interacting with TRIF and inhibiting TRIF- and TLR3-mediated activation of NF-κB, IFN-stimulated response element, and the IFN-β promoter	(77)
WWP2	HECT E3	TRIF	Promoting K48-linked ubiquitination of TRIF and degradation, inhibiting TLR3-mediated NF-κB and IRF 3 activation	(73)
HECTD3	HECT E3	TRAF3	Promoting K63-linked ubiquitination of TRAF3 and type IFN-I	(74)
NEDD4l	HECT E3	TRAF3	catalyzing K29-linked ubiquitination of TRAF3 and promoting LPS-induced IFN-I	(75)

#### 3.1.2 Role of E3 ligase in NLR signaling

**Figure 2:**
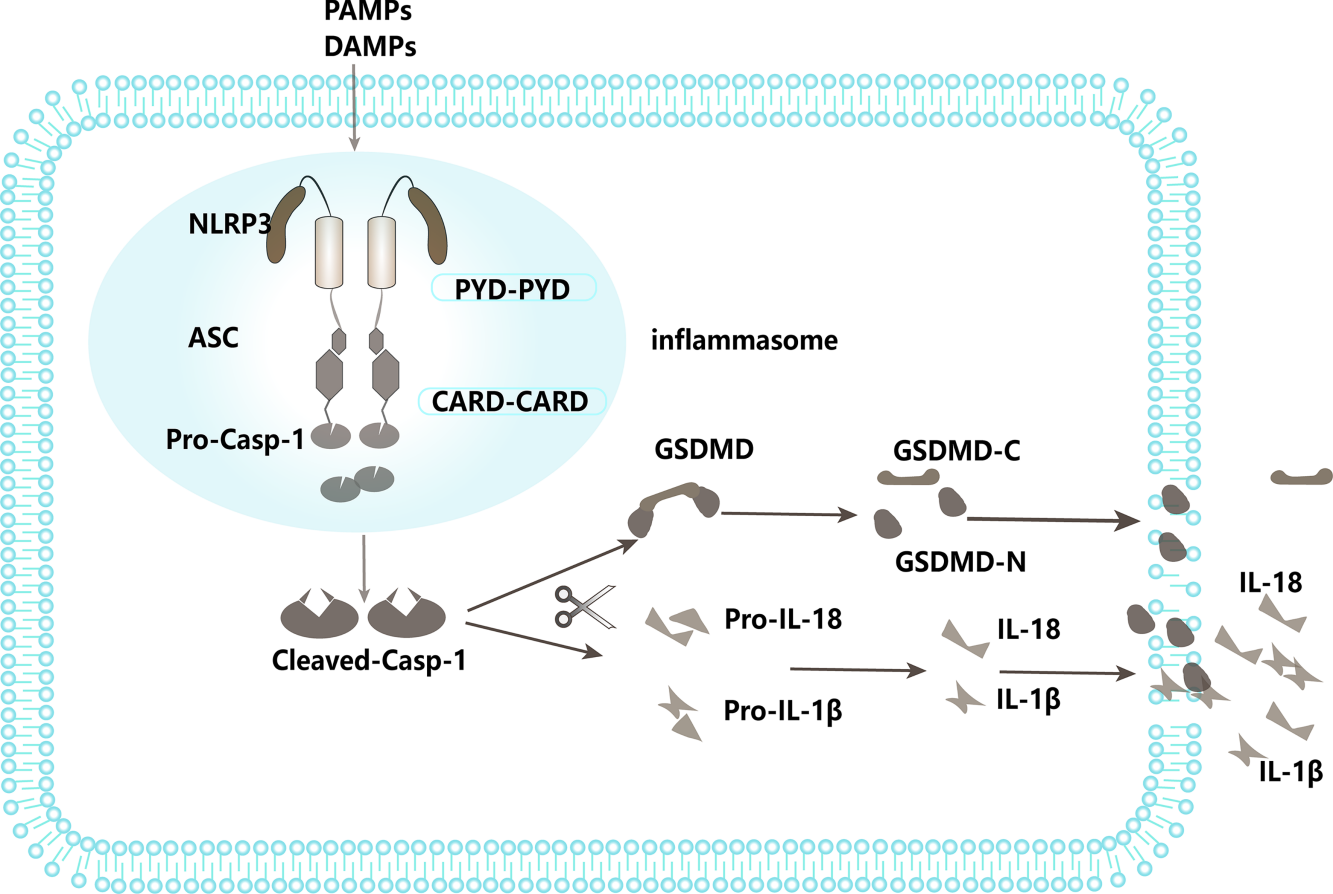
NLRP3 inflammasome pathway.

NLRs are intracellular PRRs, composed of three domains: one is the central nucleotide-binding domain (NBD), which is shared by the NLR family and is very important for nucleic acid binding and oligomerization of NLRs; LRRs at the C-terminus, which are used to identify ligands; and the N-terminal effector domain, which is the protein interaction domain, such as the caspase activation and recruitment domain (CARD) or the pyrin domain (PYD) (78). Based on the presence of an N-terminal PYD or CARD, the family is further divided into NLRP or NLRC subfamily (79). When stimulated by infection or other factors, most NLRs, including NLRP1 and NLRP3, recruit an adaptor protein, the apoptosis-associated speck-like protein containing a CARD domain (ASC), and the effector protein, pro-caspase-1, forming a large complex known as an inflammasome, resulting in the cleavage and activation of pro-caspase-1. Activated caspase-1 further cleaves IL-18 and IL-1β precursors into mature IL-18 and IL-1β, thus promoting inflammatory responses (80, 81). On the other hand, active caspase-1 also cleaves gasdermin D to free its N-terminal domain and induce the formation of pores at the membrane, leading to a pro-inflammatory form of cell death called pyroptosis (82) (**Figure 2**). This process is called canonical NLPR3 inflammasome activation (83). In addition, recent evidence points to another process named noncanonical inflammasome activation, in which LPS derived from Gram-negative bacteria stimulates caspase-11, and induces pyroptosis as well as caspase-1-dependent maturation and production of IL-1β and IL-18 (84, 85). By stimulating both innate and adaptive immune responses, activation of inflammasomes serves an important role in pathogen defense. However, dysregulation of inflammasome activity has been implicated in numerous human diseases, such as sepsis, gout, diabetes, and atherosclerosis, amongst others (86–88). For instance, studies have shown that NLRP3 inflammasome expression and activation are significantly enhanced in the peripheral blood monocytes of septic patients (89, 90). The knockout of NLRP3 or inhibition of its activation significantly reduces the release of inflammatory mediators, decreases multiple organ failure (MOF) in sepsis, and improves the survival rate of sepsis (83, 91, 92). Conversely, upregulation of NLRP3 inflammasome activation can aggravate MOF in sepsis and increase sepsis mortality (93). Previous studies also confirmed that regulation of NLRP3 inflammasome activation can affect the occurrence and development of sepsis (94, 95). Therefore, the NLRP3 inflammasome plays a key role in sepsis, and regulation of its activation can affect the progression of sepsis. Recently, an increasing number of studies have found that E3 ligases are involved in the pathogenesis of sepsis by regulating the activation of the NLRP3 inflammasome through catalyzing ubiquitination of NLRP3 or other components of the inflammasome (**Table 2**). It has been reported that the E3 ligase TRIM family plays important roles in regulating the inflammasome signaling pathway (102). For example, TRIM30 is found to negatively regulate NLRP3-mediated caspase-1activation and IL-1β production in a reactive oxygen species (ROS)-dependent manner (103).TRIM31 is also demonstrated to negatively regulate NLRP3 inflammasome signaling by promoting K48-linked polyubiquitination and subsequent proteasomal degradation of NLRP3 (96). In this study, deletion or inhibition of TRIM31 enhanced caspase-1 cleavage in ATP-treated LPS-induced mouse peritoneal macrophages and increased serum IL-1β concentration, suggesting that TRIM31 specifically attenuated NLRP3 inflammasome activation *in vivo (* 96). Additionally, TRIM65 promotes K48 and K63-linked ubiquitination of NLRP3 and inhibits NEK7 (NIMA (never in mitosis gene a)-related kinase 7)-NLRP3 interactions, leading to failure of inflammasome assembly. Consequently, caspase -1 activation and IL-1β secretion were inhibited (99). However, some TRIM members facilitate NLRP3 inflammasome activation. For example, TRIM16 and TRIM20 are demonstrated to promote IL-1β secretion through the binding to NLRP1, caspase-1, and pro- IL-1β (104–108).

**Table 2:** E3 ligases act on substrates or signaling pathways to regulate NLRP3 inflammasome.

E3	Family	Substrate/signal pathway	Function	References
TRIM31	RING E3	NLRP3	Promoting K48-linked ubiquitination of NLRP3 and degradation	(96)
FBXL2	RING E3	NLRP3	Promoting K48-linked ubiquitination of NLRP3 and reducing inflammasome activation	(97)
MARCH7	RING E3	NLRP3	Promoting ubiquitination of NLRP3 and inhibiting inflammasome activation	(98)
TRIM65	RING E3	NLRP3	Promoting ubiquitination of NLRP3 and negatively regulating Inflammasome activation	(99)
RNF125	RING E3	NLRP3	Promoting K63-linked ubiquitination of the LRR structural domain of NLRP3.	(100)
Cbl-b	RING E3	NLRP3	Promoting K48-linked ubiquitination and proteasomal pathway degradation	(100)
ARIH2	RBR E3	NLRP3	Inhibition of NLRP3 inflammasome assembly and activation	(44)
TRIM28	RING E3	NLRP3	Promoting SUMOylation of NLRP3, enhancing the stability and inflammasome activity of NLRP3	(101)

In addition to the TRIM family, other E3 ligases have been reported as endogenous negative regulators of NLRP3 inflammasome activation. The Skp-Cullin-F box (SCF) family member, F-box L2 (FBXL2), as an E3 ligase, causes K48-linked ubiquitination and proteasomal degradation of NLRP3, reducing inflammasome activation (97). Cbl-b binds to the K63-ubiquitin chain and then targets NLRP3 at K496 to catalyze K48-linked ubiquitination and proteasomal degradation (100); MARCH7 promotes K48-linked ubiquitination and autophagic degradation of NLRP3 (98); RNF125 induces K63-linked ubiquitination of NLRP3 (100). Moreover, Ariadne homolog 2 (ARIH2) ubiquitinates the NBD domain of NLRP3 and inhibits NLRP3 inflammasome activation (44). However, a recent study reported that Pellino2 promotes NLRP3 inflammasome activation by inducing the K63-linked ubiquitination of NLRP3 during the priming phase, in contrast to the inhibitory role of NLRP3 ubiquitination (109).

E3 ligases can also regulate NLRP3 inflammasome signaling by targeting other components of the inflammasome or the upstream proteins of the inflammasome. Following viral infection, MAVS (mitochondrial antiviral signaling protein)-mediated E3 ligase TRAF3 recruitment leads to K63 polyubiquitination of ASC via autophagy, leading to its degradation (110). E3 ligase can also mediate the ubiquitination of caspase-1 to regulate the activation of NLRP3 inflammasomes (111). By directly binding to DHX33 which is a cytosolic dsRNA sensor to activate NLRP3 inflammasome signaling upon dsRNA stimulation, TRIM33 induces K63-linked ubiquitination of it at K218, which is crucial for the formation of the DHX33-NLRP3 inflammasome complex, thereby leading to NLRP3 inflammasome signaling activation (112, 113).

Thus, the E3 ligases serve positive and negative roles in the activation of inflammasomes by acting on various components of the NLRP3 inflammasome, depending on cell location, the nature of the substrate, and the different ubiquitin chains that modify it.

### 3.2 Roles of E3 ligase in sepsis by regulating the adaptive immune response

When a pathogen invades the body, in addition to activation of the innate immune response, the adaptive immune response is also triggered. Innate immune cells with the ability to process and present antigens (antigen-presenting cells: APCs), in particular dendritic cells(DCs), form a link to the adaptive immune system. These inform the adaptive immune cells of danger, on the need to generate a specific immune response against the pathogen, and also provide essential initiating signals for these responses (114). The adaptive immune system consists of T and B lymphocytes characterized by the presence of highly specific antigen-recognition receptors, T-cell receptor (TCR) and B cell receptor (BCR) respectively. The activation of CD4^+^ T cells leads to the differentiation of these cells into specific T helper (Th) subsets including Th1, Th2, Th17, and follicular T (Tfh) cells, as well as the immunosuppressive regulatory T (Treg) cells (115, 116). Th1 cells secrete IL-2 to further expand memory T-cells and initiate CD8^+^T cell activation, and produce IFN-γ to promote phagocytosis and microbial eradication (117). Th2 releases IL-4 and IL-5, which act to induce class switching of B cells, and IL-10 to alleviate inflammation (118). The balance of crosstalk between Th1 and Th2 is important for clearing infections. The balance of crosstalk between Th1 and Th2 is important for clearing infections. However, when this balance is disrupted, as in the case of sepsis, it can lead to viral reactivation and secondary infections (119). Under normal conditions, both Th1 and Th2 naturally produce IL-3, which stimulates the production of granulocytes, DCs, and macrophages (120). Effector Th17 cells specifically respond to bacterial and extracellular fungal pathogens and produce cytokines such as TNF-α, IL-17, and IL-22 (121). Tregs, which represent less than 10% of the total CD4^+^ T cell population in the lymph nodes and circulation, have a critical role in immune cell modulation in both steady-state and disease settings. Tregs respond to infection by suppressing excessive immune responses elicited by other cells of the adaptive immune system, in turn, dampening inflammation (122). In response to infection, CD8^+^ T cells aid in the clearance of infection and are important for the generation of memory CD8^+^ T cells (123). B cells are at the center of the adaptive immune response where they mediate the production of antigen-specific antibodies against specific pathogens. However, similarly to T-cells, B cells also become dysregulated and exhausted as the infection spreads to become systemic (122). In sepsis, these processes are substantially disturbed, and as a result, cells of the adaptive immune system are unable to produce an appropriate defense response against the infection (122). For example, studies showed that the numbers and function of CD4^+^ T cells are greatly reduced following sepsis onset (122, 124–130). Similar to CD4^+^ T cells, CD8^+^ cells, and B cells also showed decreased ability to prevent infection and produce Ag-specific antibodies (131–134). Because T-cells serve as central components of adaptive immunity, it is not surprising that tight regulations are involved in T-cell activation and functions. Ubiquitination is an important mechanism that regulates T-cell activation and immune responses (135). Several E3 ligases are important for regulating T-cell activation and differentiation.

#### 3.2.1 Regulation of T-cell activation by E3 ligase

T-cells are derived from the bone marrow, mature in the thymus, and migrate to peripheral lymphoid tissues to perform specific cellular immune responses. Upon stimulation by an antigen, naive T-cells are activated to proliferate and subsequently differentiate into various effector T-cells that participate in different aspects of immune functions (136). The full activation of T-cells requires two distinct signals, the stimulation of TCRs by the MHC-peptide complex and co-stimulatory molecules, especially CD28, which finally initiates the downstream signaling cascades and of T-cell activation and proliferation (102). The first essential signal is delivered via the TCR/CD3 complex at the cell surface and it is related to antigen-specificity. TCR signaling initiates from the activation of the protein tyrosine kinase Lck, which phosphorylates the TCR-signaling chain CD3ζ, leading to the recruitment of the tyrosine kinase Zap70 to the TCR complex, in which Zap70 is phosphorylated and activated by Lck (136). Activated Zap70 induces the activation of several substrates, including LAT and SLP-76, to transduce the signal to PLCγ1, and downstream PI3K-AKT, MAPK, NF-κB, and calcium-dependent signaling pathways. All these signaling events finally result in the boost of transcriptional activity of NF-κB, AP1, and NF-AT, which induce gene expression for T-cell activation, and proliferation (137). The second signal is mediated by the interaction between surface co-stimulatory receptors on T-cells and their cognate ligands on the APCs (138). The co-stimulatory signal acts to amplify the signaling triggered by TCR engagement to facilitate efficient intracellular signaling (139). Lack of co-stimulatory signaling results in a state of T-cell unresponsiveness or anergy (140).

Cbl-b, a RING E3 ligase, has been identified as a critical regulator of adaptive immune responses (141). Cbl-b displays a high level of expression in CD4+ and CD8+ T cells, and functions as a gatekeeper that prohibits excessive T cell activation (142). Loss of Cbl-b in naive T cells uncouples TCR stimulation from the requirement of CD28 co-stimulation for effective proliferation and IL-2 secretion (143, 144). Additionally, Cbl-b significantly dampens T-cell activation through interaction with essential TCR signalosome molecules such as Lck, SLP76, Zap70, PKC, and PI3K (143), and the ubiquitination of some of these signalosome components such as the p85 regulatory subunit of PI3K and PLCγ1 (145). Studies show the E3 ligase GRAIL suppresses the surface expression of the TCR/CD3 complex via poly-ubiquitinating and proteasomal degradation of CD3 (146), thus inhibiting T-cell activation. GRAIL-deficient T-cells exhibit increased proliferation and cytokine secretion that is not dependent on CD28 co-stimulation (23, 146).E3 ligase NRDP1 also dampens TCR signaling. This notion is supported by the fact that CD8^+^ T cells derived from NRDP1^+/+^ mice exhibit an increased capacity to proliferate and generate IL-2 and IFN (147). ITCH, a member of the HECT-type family of E3 ligases, has also been shown to negatively regulate TCR signaling by inducing K33-linked polyubiquitylation of TCR- ζ, and subsequently attenuating the interaction between TCR- ζ chain and Zap70 (148). Additionally, ITCH suppresses NF-κB-mediated T-cell activation (149). In addition to the E3 ligase mentioned above, many other studies showed that E3 ligases have been implicated in T-cell activation and inflammation in sepsis as listed in **Table 3**.

**Table 3:** E3 ligases act on substrates or signaling pathways to regulate T-cell activation.

E3	Family	Substrate/signal pathway	Function	References
Cbl-b	RING E3	TCR and TCR signalosome molecules	Promoting ubiquitination of TCR signalosome molecules and dampening T-cell activation; Increasing TCR internalization and controlling TCR endocytosis and stability on the cell surface; Weakening TCR signalling by disrupting immune synapse activation and clustering.	(143, 145, 150, 151, 41)
ITCH	HECT E3	TCR; CBM	Promoting K33-linked ubiquitination of TCR and dampening T-cell activation; Attenuating NF-κB-mediated T-cell activation via Promoting ubiquitination of CBM and lysosomal degradation	(150, 149)
GRAIL	RING E3	TCR/CD3 complex	Activating RhoGDI through non-degrading ubiquitin chains and hindering T-cell activation; Promoting ubiquitination of CD3 and degradation	(152, 146)
NRDP1	RING E3	ZAP70	Attaching non-degradative K33-polyubiquitin chains to the ZAP70 kinase and dampening TCR signalling	(147)
Pellino 1	RING E3	c-Rel	Negatively regulating c-Rel by mediating its K48-linked ubiquitination and inhibiting T-cell activation	(153)
NEDD4	HECT E3	CBM	Attenuating NF-κB-mediated T-cell activation via Promoting ubiquitination of CBM and lysosomal degradation	(149)
MDM2	RING E3	NFATc2	Limiting CD4^+^T activation by inhibiting NFATc2 activation and cytokine production	(154)
TRAF6	RING E3	LAT	Enhancing LAT phosphorylation and TCR signalling	(155)

#### 3.2.2 Effect of E3 ligase on T-cell differentiation

T-cell differentiation is a critical step in the immune system’s fight against infections and pathogens. E3 ligases regulate T-cell differentiation by catalyzing the ubiquitination of key and specific transcription factors (**Table 4**). Transcription factor Foxp3 is expressed in almost Treg cells and is crucial for the inhibitory activity of Treg. The stability of Foxp3 directly affects the function of Tregs. Studies showed that proinflammatory cytokines and lipopolysaccharide lead to the degradation of Foxp3 through the action of the E3 ubiquitin ligase Stub1, resulting in the loss of Foxp3 and the increase of Th1 (163). Additionally, lack of E3 ligase GRAIL resulted in decreased inhibitory function in Treg cells, which was most likely related to increased expression of Th17-related genes. Studies demonstrated that GRAIL inhibits IL-21 production and Th17-specific gene upregulation in Treg cells by controlling NFATc1 expression (146). S-phase kinase-associated protein 2 (Skp2) is an important component of the SCF ubiquitin ligase complex. Skp2 can lead to the transformation between autoreactive pathogenic T cells (Tpaths) and Tregs. When Skp2 is down-regulated, there will be more regulatory T-cells. Conversely, overexpression of Skp2 leads to the loss of Foxp3 and inhibits the function of natural Treg produced in the thymus (157). On the one hand, effector T cells such as Th1, Th2, and Th17 protect against infection; on the other hand, they can cause inflammation when their function goes out of control. IL-12 promotes Th1 differentiation through signal transducers and transcriptional activators (STAT4). The E3 ligase SLIM (STAT Interacting LIM protein) catalyzes the ubiquitination and degradation of STAT protein, and also enhances the dephosphorylation of STAT4 and inhibits its activity. SLIM deficiency leads to increased STAT expression, Th1 differentiation, and IFN-γ production (158). Inducible T-cell co-stimulators (ICOS) are induced after T-cell activation and are members of the CD28 family. Overexpression of E3 ligase Roquin leads to upregulation of CD28 and down-regulation of ICOS when inhibiting T-cell activation. By regulating CD28/ICOS signaling, Roquin increases Th1 cells and the secretion of IL-2 and pro-inflammatory cytokines (159). ITCH regulates Th2 differentiation by catalyzing JunB ubiquitination. Consistent with this, studies show that JunB activity and secretion of IL-4 and IL-5 increased in ITCH ^-/-^ T cells. As we know the transcription factor JunB is responsible for IL-4 expression that can promote the Th2 cell differentiation (160). The E3 ligase Act1 promotes the production of Th2 cytokine IL-25. Act1-deficient T-cells induce attenuation of key Th2-related transcription factors GATA-3 and GFI-1 and are less responsive to IL-25-driven differentiation into Th2 (161). Transcription factor Rorc is responsible for the expression of IL-17 and induces Th17 differentiation. Phosphorylated signal transducer and activator of transcription 3 (STAT3) were activated to induce transcription of Rorc. The E3 ligase PDLIM2 catalyzes the ubiquitination and degradation of STAT3, inhibits STAT3-mediated gene activation, and attenuates Th17 cell differentiation. PDLIM2-deficient T-cells showed increased STAT3 production and enhanced Th17 cell differentiation (164). E3 ligase TRIM21 catalyzes ubiquitination of interferon regulatory factor (IRF), reducing the secretion of IRF-regulated cytokines such as IL-6, IL-12/IL-23p40, and IL-17, all of which are involved in the Th17 pathway.

**Table 4:** E3 ligases act on substrates or signaling Pathways to Regulate T-cell differentiation.

E3	Family	Substrate/signal pathway	Function	References
GRAIL	RING E3	TCR/CD3 complex	Targeting the TCR-CD3 complex for degradation restricts NFATc1 expression; inhibiting IL-21 production and upregulation of Th17-specific genes in Treg cells	(146)
Cbl-b	RING E3	PI3-K	Regulating the development and function of iT regs via modulating PI3K–Akt–Foxo3a signalling	(156)
Skp2	RING E3	/	Decreasing Foxp3 expression and abrogating the suppressive function of nTregs	(157)
SLIM	RING E3	STAT proteins	Controlling the extent of STAT-mediated Th1 cell differentiation and IFN-γ production following an inflammatory response	(158)
Roquin	RING E3	/	Increasing Th1 cells and the secretion of IL-2 and pro-inflammatory cytokines by regulating CD28/ICOS signalling,	(159)
ITCH	HECT E3	Transcription factor JunB	Regulating Th2 differentiation by catalysing JunB ubiquitination	(160)
Act1	U-box type E3	/	Promoting the production of Th2 cytokine IL-25	(161)
PDLIM2	RING E3	STAT3	Promoting ubiquitination of STAT3 and attenuating Th17 cell differentiation	(161)
TRIM21	RING E3	IRF	Reducing the secretion of IRF-regulated cytokines via catalysing ubiquitination of IRF and attenuating Th17 cell differentiation	(162)

## 4 Conclusion

Sepsis is characterized by dysregulated host innate and adaptive immune response. Better understanding molecular mechanisms that govern inflammatory and immune-associated cellular functions is important and urgent. Ubiquitination plays a pivotal role in regulating immune responses. In theory, the most valid ubiquitination-directed treatment is blocking E3 ligases in specific substrate recognition sites. In this review, we highlight the functions of E3 ligases in the regulation of innate and adaptive immune responses. In recent years, a large number of E3 ligases have been characterized as important regulators in immune response, ranging from PRR signaling in the innate immune system to T cell activation and differentiation in sepsis. Given the importance of E3 ligases in the regulation of immune response in sepsis, deeper insights into the roles of E3 ligases in regulating immune response probably reveal novel therapeutic targets for the treatment of sepsis.

## Author contributions

DQZ, JF, and SS contributed to conception and design of the study. YL and HY organized the database. SS and DQZ wrote the first draft of the manuscript. JF, DXZ and B wrote sections of the manuscript. DXZ created relevant figures and tables. All authors contributed to the article and approved the submitted version.
